# PET-Guided Surgery — High Correlation between Positron Emission Tomography with ^11^C-5-Hydroxytryptophane (5-HTP) and Surgical Findings in Abdominal Neuroendocrine Tumours

**DOI:** 10.3390/cancers4010100

**Published:** 2012-02-08

**Authors:** Håkan Örlefors, Anders Sundin, Barbro Eriksson, Britt Skogseid, Kjell Öberg, Göran Åkerström, Per Hellman

**Affiliations:** 1 Departments of Medical Sciences, Uppsala University, Uppsala SE-751 85, Sweden; E-Mails: hakan.orlefors@akademiska.se (H.O.); barbro.eriksson@medsci.uu.se (B.E.); britt.skogseid@medsci.uu.se (B.S.); kjell.oberg@medsci.uu.se (K.O.); 2 Department of Radiology, Karolinska Hospital, Institution of Molecular Medicine and Surgery, Karolinska Institute, Stockholm SE-171 77, Sweden; E-Mail: anders.sundin@ki.se; 3 Department of Radiology, Uppsala University Hospital, Uppsala SE-751 85, Sweden; 4 Department of Surgical Sciences, Uppsala University, Uppsala SE-751 85, Sweden; E-Mail: goran.akerstrom@surgsci.uu.se

**Keywords:** neuroendocrine tumour, positron emission tomography, hydroxytryptophane, insulinoma, surgery

## Abstract

Positron emission tomography (PET) with ^11^C-labeled 5-hydroxytryptophane (5-HTP) is a sensitive technique to visualize neuroendocrine tumours (NETs), due to high intracellular uptake of amine-precursors like L-dihydroxyphenylalanine (L-DOPA) and 5-HTP. NETs are often small and difficult to localize in spite of overt clinical symptoms due to hormonal excess. In our study, 38 consecutive NET patients underwent ^11^C-5-HTP-PET and morphological imaging by CT within 12 weeks prior to surgery. Surgical, histopathological and 5-HTP PET findings were correlated. ^11^C-5-HTP-PET corresponded to the surgical findings in 31 cases, was false negative in six, and true negative in one case resulting in 83.8% sensitivity and 100% specificity. Positive predicted value was 100%. In 11 patients ^11^C-5-HTP-PET was the only imaging method applied to localize the tumour. Thus, we could demonstrate that functional imaging by ^11^C-5-HTP-PET in many cases adds vital preoperative diagnostic information and in more than every fourth patient was the only imaging method that will guide the surgeon in finding the NET-lesion. Although the present results demonstrates that ^11^C-5-HTP may be used as an universal NET tracer, the sensitivity to visualize benign insulinomas and non functioning pancreatic NETs was lower.

## 1. Introduction

Visualization of the tumour is crucial in the preoperative work-up of patients with neuroendocrine tumours (NETs). Despite pronounced clinical symptoms and elevated hormone-markers in peripheral blood the NET-lesions can be small and situated almost anywhere in the body. This tumour entity is greatly heterogeneous, with vast biochemical differences, and great variations in proliferation rates and malignant potential. Although NETs are generally well differentiated and display a low grade of proliferation the tumours have nevertheless in many cases already spread at the time of diagnosis [1]. In addtion, the clinical syndrome may not appear until metastases are present. For instance, patients with small intestinal NETs and the carcinoid syndrome often have liver metastases at the time of diagnosis [1]. Pancreatic NETs may be benign without signs of dissemination, but still cause severe clinical symptoms related to hormone excess. There are also malignant variants of pancreatic NETs, with local invasion and metastases to regional lymph nodes and the liver [2]. Another clinical diagnostic problem is the pancreatic tumours in multiple endocrine neoplasia type 1 (MEN-1), the presence of which sometimes is indicated merely by biochemical signs [3].

Besides localization of the primary tumour prior to surgery, staging of the disease is a prerequisite for therapy planning [1,3]. Taken together, there are needs for sensitive and specific imaging methods for reliable lesion detection and scheduling of the treatment.

Positron emission tomography (PET) with the tracer ^18^F-fluorodeoxyglucose (FDG), has become a powerful method for imaging of most types of cancer. Malignant tumours may be detected by FDG-PET with high efficacy due to the increased utilization of glucose in these tumours as compared to most normal tissues. This has been shown to be true also in NETs with high proliferation-rate. However, in well-differentiated NETs, the FDG uptake is generally low, making this technique of limited value [4,5].

Anatomical imaging by computed tomography (CT) and magnetic resonance imaging (MRI) still forms the basis for tumour visualization, but cannot always visualize smaller tumours [6,7]. Functional imaging by somatostatin receptor scintigraphy (SRS) has clearly demonstrated its usefulness for whole body scanning and staging of NETs, and as a tool for evaluation before therapy with somatostatin analogues or peptide receptor radionuclide therapy (PRRT) [8,9]. However, SRS has its limitation due to the restricted spatial resolution at planar imaging and single-photon-emission tomography (SPECT) and the variable affinity and expression of somatostatin receptors [10,11]. Recently, preparations of ^68^Ga-labeled somatostatin analogs have been developed for somatostatin receptor imaging by PET to utilize the better spatial resolution of PET as compared to SRS. This new technique has shown higher sensitivity than SRS to visualize various NETs [12].

PET using the ^11^C-labeled serotonin-precursor 5-hydroxytryptophane (5-HTP) has been shown to be a very sensitive modality for NET imaging that surpassing that of both SRS and CT [13,14,15]. 5-HTP-PET seems to be useful in most tumour entities within the heterogeneous NET group, most likely attributed to the amine-precursor uptake mechanisms generally present in NET cells. Thus, precursors like 5-HTP and L-dihydroxyphenylalanine (L-DOPA) can be internalized into the NET cell and thereby incorporated in the synthesis of peptide hormones like serotonin (5-HT) and dopamine.

In order to evaluate the role of preoperative ^11^C-5-HTP PET as a tool for surgical assistance in tumour detection, and to compare this imaging technique to “gold standard” for tumour localization, *i.e*., surgery, we evaluated ^11^C-5-HTP PET imaging in patients with abdominal NETs subjected to operation a short time after the examination.

## 2. Results and Discussion

### 2.1. Overall Results 5-HTP PET

**Figure 1:**
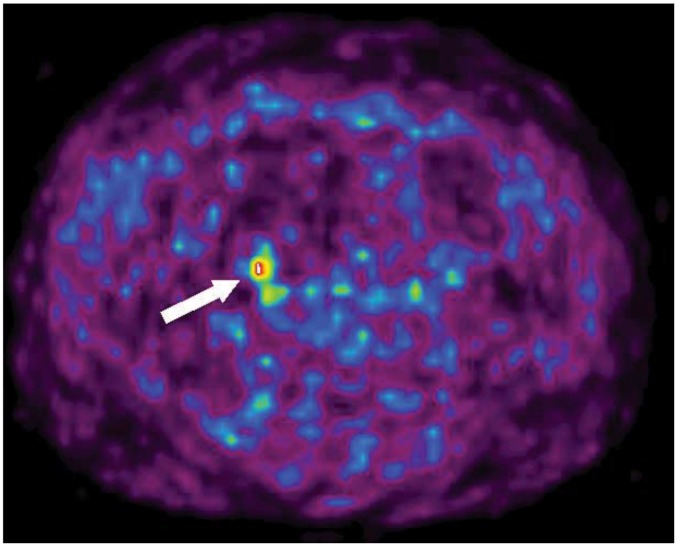
Transaxial ^11^C-5-HTP PET image of a patient with small intestinal NET (patient 16). A suspicious metastatic lymph node metastasis is seen (arrow). It was later confirmed at histopathology.

**Figure 2:**
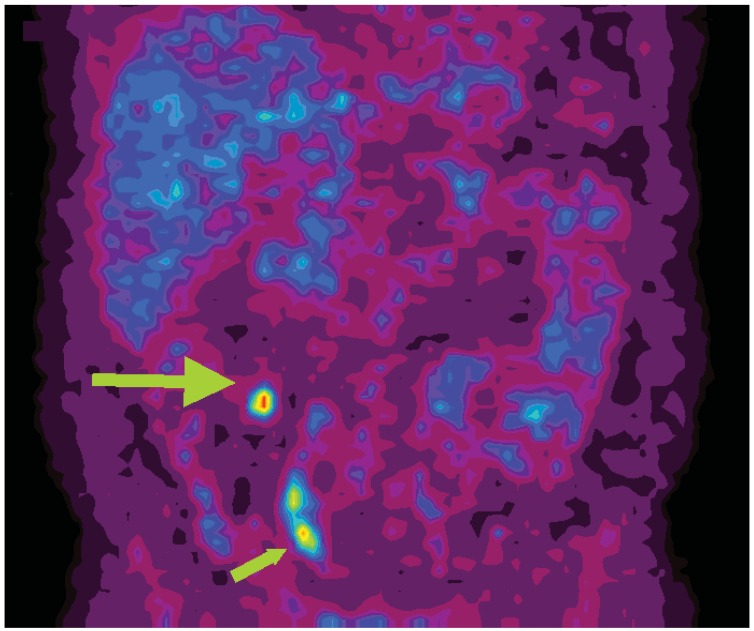
Coronal ^11^C-5-HTP PET image of a patient with small intestinal NET (patient 30). A focal high tracer uptake (large arrow) represents a mesenteric lymph node metastasis. Also urinary radioactivity is seen in the right ureter (small arrow).

**Figure 3:**
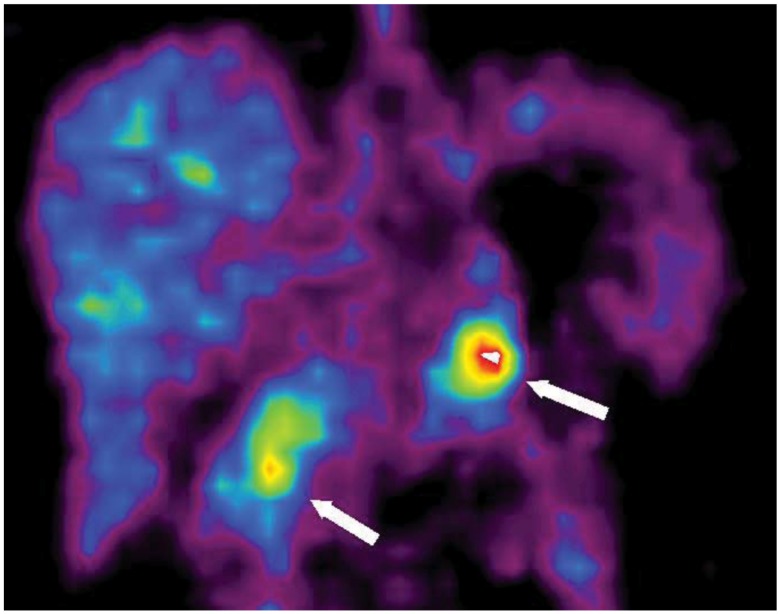
Coronal ^11^C-5-HTP PET image of a patient with a MEN-1 syndrome and biochemical signs of a pancreatic tumor (patient 2). Two pancreatic tumors, one in the head and one in the tail, were seen at PET and were both later confirmed by surgery. The tumors in the head as well as the tail of the pancreas are clearly depicted (arrows: to the right = pancreatic head; to the left = pancreatic tail).

**Figure 4:**
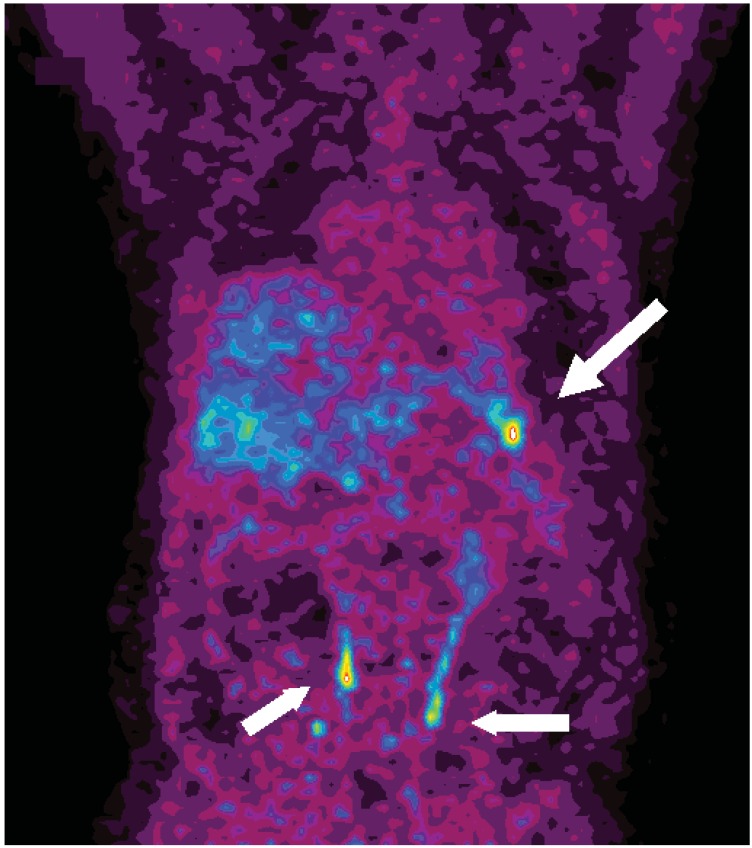
Coronal ^11^C-5-HTP PET image of a patient with a MEN-1 syndrome and an NET in the pancreatic tail (arrow, patient 32). Urinary radioactivity is seen in both ureters (shorter arrows).

5-HTP PET visualized tumours in 31 patients (Figure 1, Figure 2, Figure 3, Figure 4). In all these cases histopathologically proven NETs were found in the corresponding anatomical sites. In seven patients PET was negative, although NETs were found at surgery in six of these patients; one patient had a negative exploration and a truly negative PET. PET thus corresponded anatomically to the surgical findings in 31 cases, were false negative in six, and true negative in one case (case 10, see description in Experimental Section). Overall sensitivity was 83.8%, specificity 100%, positive predicted value 100% and negative predicted value 14.3%.

In 11 patients ^11^C-5-HTP-PET was the only imaging method that was able to localize the tumour, as illustrated in Figure 1 by one patient with small intestinal NET disease and an apparent primary tumour in the small intestine. At CT no mesenteric lymph node metastasis was found in a fatty mesentery but by 5-HTP PET a small 8 mm mesenteric lymph node metastasis was readily depicted which later was surgically and histopathologically confirmed (Figure 1). Tumours visible on only PET were all less than 20 mm in size (mean 10 mm). In most patients PET was positive also in other than the resected areas. The tracer uptakes corresponded in a majority of these patients to presence of liver metastases, documented at the abdominal exploration but not always resected at operation. The tumours missed on PET but later confirmed by surgery and histopathology were all 10 mm or less in diameter. In five cases tumours less than 10 mm were visible by PET.

### 2.2. Different Types of NETs and 5-HTP PET

When the results were assessed in relation to tumour type, sensitivity for 5-HTP PET for detection of insulinomas was lower (Table 1). Out of six patients with insulinomas, only two demonstrated unequivocal localization by PET that corresponded to the surgical findings. Two patients were negative on PET; one patient demonstrated a slightly higher uptake in the pancreatic head while the tumour was rather located in the pancreatic body. In one patient a metastasis in the pancreatic tail was visualized while PET failed to diagnose the primary tumour in the head.

**Table 1:** List of diagnoses and ^11^C-5-HTP PET results in correlation to findings at surgery and histopathology.

Diagnos	n	True pos	False neg	True neg
SI-NET	10	10		
NF PNET	6	4	2	
Insulinoma	6	4	2	
Gastrinoma	3	3		
Gluc-prod PNET	2	2		
ACTH-prod PNET	2	2		
MEN-1 PNET	7	5	2	
Pheochromocytoma	1	1		
Type III gastric NET	1			1

Abbreviations: SI-NET = Small intestinal NET; NF PNET = Non-functioning pancreatic NET; Gluc-prod PNET = glucagon-producing pancreatic NET; ACTH-prod PNET = ACTH-producing pancreatic NET.

5-HTP PET visualized all four patients with gastrinomas, one of them as part of MEN-1, although in two of the patients a tumour was never histopathologically proven at surgery, but still classified as true positive. In one of these patients a corresponding 3 mm non-resected intrapancreatic lesion in the head, and in the other a 8 mm lesion in the tail were demonstrated by intraoperative ultrasound (patients 5,17). Non-functioning pancreatic NETs were also difficult to visualize by PET, which in two out of six patients were false negative. By contrast, in patients with small intestinal NET all tumours were visualized by PET, and later confirmed by surgery and histopathology (Figure 1, Figure 2).

### 2.3. Discussion

In this study we have, in a considerable number of patients, demonstrated that functional imaging with ^11^C-5-HTP-PET in many cases add vital preoperative diagnostic information and can sometimes offer crucial guidance to the surgeon in finding the NET or its metastases. In more than one fourth of the patients, PET was the only method that could localize the tumour. The sizes of these tumour lesions were small (mean 10 mm) and therefore easily overlooked by anatomical imaging methods like CT and MRI. In this sense the specific uptake of the radiolabeled tracer ^11^C-5-HTP in the tumour tissue results in a high tumour-to-background ratio that enables delineation from normal abdominal structures and thereby good tumour visualization. The present study was initiated before PET/CT was available at our institution and merely a few patients were examined by this technique. In the study design PET/CT was performed using a low dose CT for attenuation correction and was not used as a means to read the PET images. While the present study focuses on ^11^C-HTP PET and surgical findings, the impact of ^11^C-5-HPT PET/CT on the pre surgical imaging work up is currently being performed.

The mechanisms underlying the high uptake of the amine-precursor 5-HTP into the vast majority of NET cells are not fully elucidated [15]. In the literature these tumours are defined as APUD-omas (Amine Precursor Uptake and Decarboxylation) due to their ability to internalize amine precursors like 5-HTP and L-DOPA, and process them into peptide hormones like 5-HT (serotonin) and dopamine [16]. This mechanism has been demonstrated *in vivo* in NETs where intracellular uptake and decarboxylation of ^11^C-L-DOPA and ^11^C-5-HPT have been visualized by PET in patients [17]. Moreover, there is most likely an upregulation of transmembrane amino acid transporters and intracellular enzymes responsible for peptide hormone metabolism (e.g., AADC, monoamine oxidase) in these tumours, thereby contributing to the increased amine precursor uptake and turnover. As an example of higher MAO-A levels in NETs, the uptake of the MAO-A-binding molecule harmine labelled with ^11^C was increased in NETs which enhanced tumour visualization [18].

^11^C-5-HTP PET has earlier been identified as a sensitive method for detection of NETs and as a tool for evaluation of therapy. Also, a comparison of ^11^C-5-HTP and ^18^F-DOPA showed that the former tracer was better to visualize pancreatic NETs and the latter to localize small intestinal NETs, and the recent development of ^68^Ga-DOTA-somatostatin analogs as tracers for PET add several imaging possibilities to this field [13,19]. However, a strict imaging correlation to surgical findings (“gold standard”) with histopathological confirmation, as in the present study, has previously not been demonstrated. The high sensitivity of 5-HTP PET reveals itself by being the only modality that could demonstrate areas harbouring liver metastases. Thus, 5-HTP-PET may reveal additional metastases and a more disseminated disease than anticipated by other methods, which is important in a staging process and for future therapy discussion. On the other hand, this may be regarded as false positive signals, but in the lack of histopathology difficult to interpret. However, it is our clinical experience that positive PET-lesions become visualized as tumours when repeated anatomical imaging is performed over the years. This can be exemplified by patient No. 32, where preoperative ^11^C-5-HPT-PET indicated a tumour lesion in the liver. This lesion could not be verified at surgery, but nevertheless a year later a tumour lesion at the same site was found guided by abdominal ultrasonography, and biopsy-verified as a metastasis to the pancreatic NET. However, one also has to bear in mind that our patients are highly selected being referred to a tertiary center specialized in neuroendocrine tumours, which may lead to misleadingly high sensitivity numbers for 5-HTP-PET. Nevertheless, in the context of our preoperative work-up, we note that 5-HTP PET is an important and valuable tool. Another limitation is the short half-life of ^11^C-labelled compunds, leading to that 5-HTP-PET is limited to being used in centres equipped with a cyclotrone.

The highest uptake and fewest negative PET examinations were in our series seen in small intestinal NET patients, partly in contrast to Koopmans *et al*. [13]. 5-HTP is the precursor to 5-HT (serotonin), which is metabolized to 5-hydroxyindole acetic acid (5-HIAA) and measurable in the urine as the main biochemical marker for small intestinal NET. Therefore it is expected that 5-HTP PET easily visualize small intestinal NET.

However, also tumours with limited peptide-hormone production, such as non-functioning pancreatic NETs including many MEN-1 related pancreatic tumours show uptake of 5-HTP to the extent that they can be visualized by ^11^C-5-HTP-PET. Functioning pancreatic NETs producing glucagons and gastrin seem to be well imaged by ^11^C-5-HTP-PET in the present as well as previous studies [15,20]. On the other hand, insulinomas are known to be difficult to image with SRS, CT and MRI [21]. In these patients surgeons have performed laparotomy without localization and relied on intraoperative examination of the pancreas including palpation and intraoperative ultrasound. In the present study four out of six insulinomas are visualized by ^11^C-5-HTP-PET, indicating somewhat lower sensitivity than for other diagnoses, bus also promising compared to other modalities, and needs to be further elucidated in future studies. In this regard the study by Koopmans *et al.* does not clarify this matter either, since they have gathered all pancreatic NETs together without describing different characteristics important for the surgeon [13]. In addition, the finding that two patients with ectopic ACTH-production and Cushing´s syndrome as well as the patient with a pheochromocytoma are detected by ^11^C-HTP PET, proves the universal use of this technique for detection of basically all groups of NETs.

Imaging with 5-HTP PET unequivocally guided the surgical procedure in several cases. An example is one patient with small intestinal NET, where PET depicted a rather small lymph node metastasis, not immediately found at surgery, and since the location was known at PET, an extended mesenteric resection was performed and later the positive metastatic lymph node was found and histopathologically confirmed (Figure 1).

## 3. Experimental Section

In 38 consecutive patients (14 females, mean age 50 years, range 20–73) with abdominal NETs, ^11^C-5-HTP-PET was performed within 12 weeks before surgery. Eight of the patients had been operated upon previously (Pat. Nos. 4, 9, 10, 15, 21, 30, 32, 34; Table 2), and were subjected to reoperation due to persistent disease, comprising predominantly of lymph node or liver metastases. The diagnoses of the tumours are depicted in Table 2. The results of the preoperative 5-HTP-PET and CT examinations were known to the surgeons (GÅ, PH) at operation. The Local Ethics and the Radiation Protection Committees approved the study.

**Table 2:** List of patients subjected to ^11^C-5-HTP PET before surgery, short notes for the PET and intraoperative findings, and commented outcome.

Pat No.	Diagnosis	PET	Surgery	Outcome of PET TP/TN/FP/FN
1	Insulinoma	Head	Head	TP
2	MEN1	Body + tail	Body + tail	TP
3	Insulinoma	Head/Body	Head/Body	TP
4	NF PNET	0	Body	FN
5	Gastrinoma	Liver, tail	Tail	TP
6	Insulinoma	0	Tail	FN
7	Insulinoma	0	Head	FN
8	ACTH-prod PNET	Liver × 10, lgll	Liver, lgll	TP
9	Gluc-prod PNET	Lgll	Lgll	TP
10	Gastric NET	0	0	TN
11	SI-NET	Liver × >10, lgll, ribs	Int, mes, liver	TP
12	NF PNET	Body, lgll, liver	Body, lgll, liver	TP
13	Insulinoma	Head, Body	Body	TP
14	NF PNET	Tail	Tail	TP
15	SI-NET	Liver × 2, lgll	Liver × 5, lgll	TP
16	SI-NET	Mes lgll	Int, mes lgll	TP
17	Gastrinoma	Head	Head	TP
18	SI-NET	Mes	Int, mes lgll	TP
19	Pheochromocytoma	Left adr	Left adr	TP
20	MEN-1	0	Head, Body, Tail	FN
21	MEN-1	Lgll	Lgll	TP
22	SI-NET	Liver × 2, mes lgll	Liver × 2, int, mes lgll	TP
23	SI-NET	Mes, lgll, liver × 3	Int, mes lgll, liver × 2	TP
24	ACTH-prod PNET	Tail	Tail	TP
25	NF PNET	0	Body	FN
26	NF PNET	Body	Body	TP
27	Insulinoma	Tail	Head + Tail	TP
28	NF PNET	Body	Body + liver	TP
29	MEN-1 + gas	Head, lgll, gastr	Lgll, duod, gastr	TP
30	SI-NET	Mes lgll	Mes lgll	TP
31	MEN-1	0	Body	FN
32	MEN-1	Tail	Tail, lgll	TP
33	SI-NET	Liver × 10, mes lgll	Int, mes lgll, liver × >10	TP
34	Gluc-prod PNET	Liver × 2	Liver × 3	TP
35	MEN-1	Head, Body	Head, Body, liver × 2	TP
36	SI-NET	Lgll	Int, mes lgll	TP
37	Gastrinoma	Liver × 10, Head, lgll	Lgll, liver × 10	TP
38	SI-NET	Mes, liver × 2, lgll	Int, mes lgll, liver × 3	TP

Abbreviations: MEN-1 = multiple endocrine neoplasia type 1 with signs of pancreatic tumor; NF PNET = Non-functioning pancreatic NET; ACTH-prod PNET = ACTH producing pancreatic NET giving rise to ectopic Cushing’s syndrome; SI-NET = small intestinal NET; Caput = pancreatic head; corp = pancreatic body; cauda = pancreatic tail; Liver × 10 = approximately 10 metastases in the liver; Lgll = pathological lymph nodes; Mes lgll = mesenteric metastatic lymph nodes; gastr = gastric tumor; adre = adrenal gland tumor; Int = intestinal tumor; duod = duodenum; TP = true positive; TN = true negative; FP = false positive; FN = false negative.

### 3.1. Surgery

The surgical procedures were performed according to standard protocols at the Department of Surgery, University Hospital, Uppsala, Sweden. The 10 patients with small intestinal NETs underwent ileocecal resection with mesenteric lymph node clearance (n = 5) as previously described [22], or only intestinal resection in three cases. In two patients concomitant liver resections were performed. Two small intestinal NET patients had been previously operated on, and now underwent resection of mesenteric lymph node metastases without intestinal resection, and a left-sided hemihepatectomy together with resections of several liver metastases on the right side. One patient was subjected to two operations within a short time interval with an ^11^C-5-HTP PET examination performed before the first operation. This patient underwent ileocecal resection and mesenteric lymph node clearance and subsequently right hemihepatectomy and a local tumour excision in the left liver lobe.

Of the 13 sporadic patients with functioning pancreatic NETs, six had insulinomas. Two underwent pylorus-preserving pancreaticoduodenectomy, in one of them combined with distal pancreatectomy because of multiple tumours; three others were subjected to distal pancreatectomy, and one enucleation of the tumour. Three patients were operated on for gastrinoma, one with duodenectomy and resection of duodenal gastrinoma as well as resection of regional lymph node and liver metastases. Two patients underwent exploratory laparotomy without resection of tumour. Two patients previously operated with pancreatic resection for glucagonoma were reoperated with resection of lymph node metastases and resection of liver metastases. Two patients had ectopic ACTH-production; one in a pancreatic NET resected by distal pancreatectomy and another with pancreatic NET and concomitant multiple liver metastases who were subjected to bilateral adrenalectomy.

The six patients with non-functioning pancreatic NETs underwent distal pancreatectomy with splenectomy, re-resection of the pancreatic rim after previous distal pancreatectomy, enucleation, and only laparotomy (inoperable tumour).

Of seven patients with MEN-1, five with pancreatic tumours underwent subtotal pancreatectomy and splenectomy in two combined with enucleations of pancreatic head tumours. Another MEN-1 patient previously subjected to pancreatic resection underwent extirpation of regional lymph node metastases, and one patient was subjected to duodenotomy and resection of multiple duodenal gastrinomas together with near total gastric resection due to a Zollinger-Ellison syndrome with extensive type II gastric NET.

One patient in this series had a sporadic pheochromocytoma and underwent a laparoscopic left-sided adrenalectomy. One patient previously operated for type III gastric NET, now underwent exploratory laparotomy due to suspicion of lymph node metastasis according to a somatostatin receptor scintigraphy. However no pathological findings were found, implying a false positive somatostatin receptor scintigraphy, but a true negative HTP-PET.

During each operation a thorough evaluation was performed of the area described by 5-HTP PET and CT as being positive for tumour, and surgical findings were documented and compared to the radiological findings. Extirpated tumours were analyzed by histopathology for confirmation of endocrine differentiation. This was performed by chromogranin A and synaptophysin immunostaining, combined with staining for the various pancreatic hormones.

### 3.2. PET

^11^C-5-HTP was synthesized according to previously described procedures [23]. One hour before PET examination carbidopa (200 mg) was administered orally in order to block the enzyme aromatic amino acid decarboxylase [24]. The patient’s body weight was 78 ± 14 (mean ± SD) kg and ranged from 53 to 103 kg. ^11^C-5-HTP at a dose of 511 ± 214 (mean ± SD, range 95–850) MBq corresponding to 6.7 ± 3.0 (mean ± SD, range 1.6–14.2) Mbq/kg body weight was injected intravenously and PET-imaging was started 20 minutes after tracer injection. Thirty-one patients were examined in a Siemens ECAT HR + PET scanner (Erlangen, Germany) with a 15.5 cm axial field of view for each bed-position, providing 63 transaxial slices with a thickness of 2.5 mm. In each bed position a 5 minutes emission was acquired together with a 10 mm transmission scan with an external rotating ^68^Germanium pin for attenuation correction. Seven patients were examined by PET/CT in a GE Discovery ST scanner (General Electric, Waukesha, WI, USA), with a 15.7-cm axial field of view providing 3.7 mm transaxial slices. A low-dose CT (140 kV, 80 mA) examination was used for attenuation correction and anatomical correlation of the PET findings. The PET examination typically comprised 5–6 bed positions and the images were reconstructed using Ordered Subset Expectation Maximization (OSEM) with standard software delivered together with the respective scanners. Evaluations of the ^11^C-5-HTP-PET and PET/CT examinations were performed by one radiologist (AS) who was blinded for the CT-findings.

### 3.3. CT

CT of the abdomen and chest was performed on a Siemens Somatom Volume Zoom 4 detector row CT scanner (Erlangen, Germany) using a standard clinical examination protocol. The acquisition was performed using 4 × 2.5 mm detectors and collimation and the images were reconstructed as 3.0 mm sections with a 2.5 mm reconstruction increment. The liver and pancreas were examined before and during intravenous contrast enhancement in the portal venous inflow phase and the venous phase. The chest and the rest of the abdomen were examined in the venous contrast enhancement phase.

## 4. Conclusions

In conclusion, we have proven that tumours diagnosed by 5-HTP PET are NETs as assessed by histopathology and immunostaining. Our results indicate that 5-HTP PET accurately describes the extent of the disease, which is crucial in the preoperative decision-making, and is a useful tool to guide the surgeon pre- and peroperatively.
